# Endoscopic ultrasound-guided fine-needle biopsy of abdominal lymphadenopathy in Kimura’s disease

**DOI:** 10.1055/a-2974-0631

**Published:** 2026-10-16

**Authors:** Songming Ding, Lihong Cao, Yang Liu, Qi Zhou, Shusen Zheng, Qiyong Li

**Affiliations:** 1 Division of Hepatobiliary and Pancreatic Surgery 636046 Shulan Hangzhou Hospital Affiliated to Zhejiang Shuren University Shulan International Medical CollegeHangzhouChina; 2 Department of Hematology 636046 Shulan Hangzhou Hospital Affiliated to Zhejiang Shuren University Shulan International Medical CollegeHangzhouChina; 3 Endoscopy Center 636046 Shulan Hangzhou Hospital Affiliated to Zhejiang Shuren University Shulan International Medical CollegeHangzhouChina

Kimura’s disease (KD) is a rare, benign, idiopathic chronic inflammatory disorder endemic to Asia that predominantly affects men in their second and third decades of life. 1​ It is classically characterized by painless, solid masses in the deep subcutaneous tissues of the head and neck—particularly involving the salivary glands and regional lymph nodes—accompanied by peripheral blood eosinophilia and elevated serum immunoglobulin E (IgE) levels. 2​ ​3​ We herein report a 62-year-old man with a 3-year history of KD, previously treated with corticosteroids, who presented with abdominal lymphadenopathy.

The patient was admitted due to a 1-year history of recurrent abnormal liver biochemistry. Laboratory investigations revealed elevated levels of aspartate aminotransferase (82 U/L), gamma-glutamyl transferase (205 U/L), alkaline phosphatase (670 U/L), and bilirubin (total 54 μmol/L and direct 41 μmol/L). The eosinophil count peaked at 5.31×10 ^9^ /L (accounting for 54% of total white blood cells), and the peripheral blood smear confirmed marked eosinophilia ( Fig. 1 ). A bone marrow biopsy further demonstrated increased eosinophil proliferation ( Fig. 1 ). Serum IgE was elevated at 119.0 KIU/L, and flow cytometry immunophenotyping detected no lymphoma clone. Imaging revealed multiple enlarged lymph nodes in the hepatic hilum, hepatogastric space, and portocaval space, accompanied by mild intrahepatic bile duct dilation ( Fig. 2 ). Positron emission tomography/computed tomography showed metabolically active lymphadenopathy consistent with KD; however, a biopsy was recommended to rule out lymphoma ( Fig. 3 ).

**Fig. 1:**
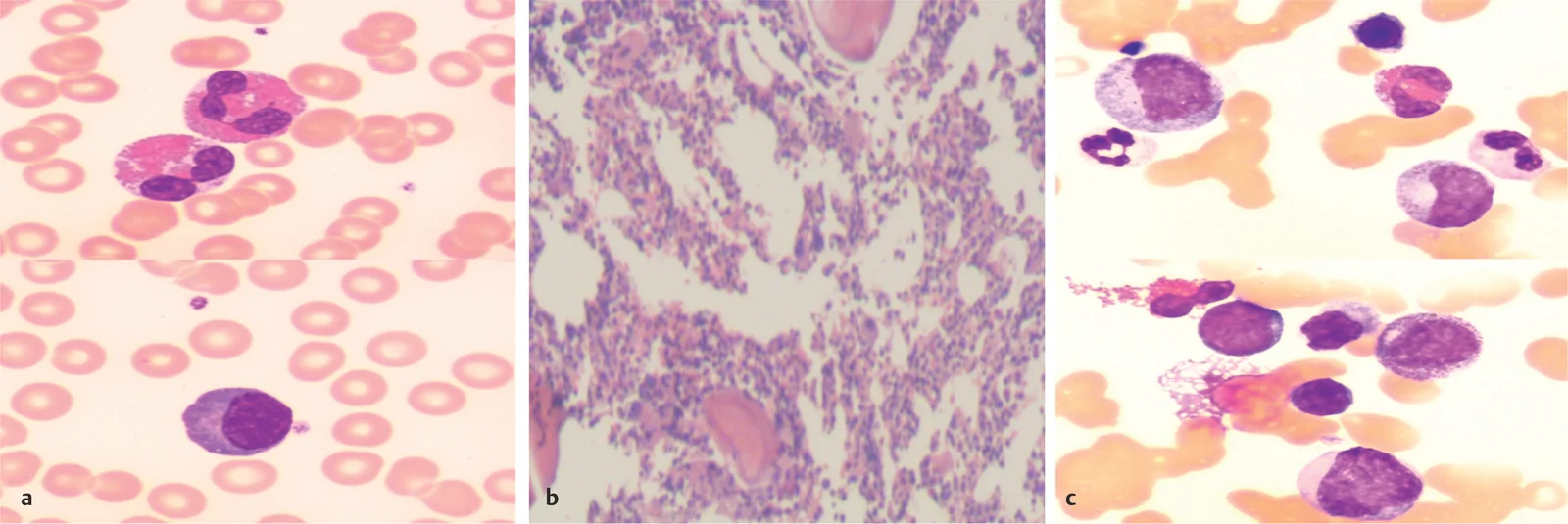
Peripheral blood smear and bone marrow biopsy. ( **a** ) The peripheral blood smear showed eosinophilia. ( **b** ) Bone marrow biopsy revealed approximately normal cellularity with proliferation of the granulocytic, erythroid, and megakaryocytic lineages, along with an increased number of eosinophils. ( **c** ) Bone marrow smear showing an increased number of eosinophils.

**Fig. 2:**
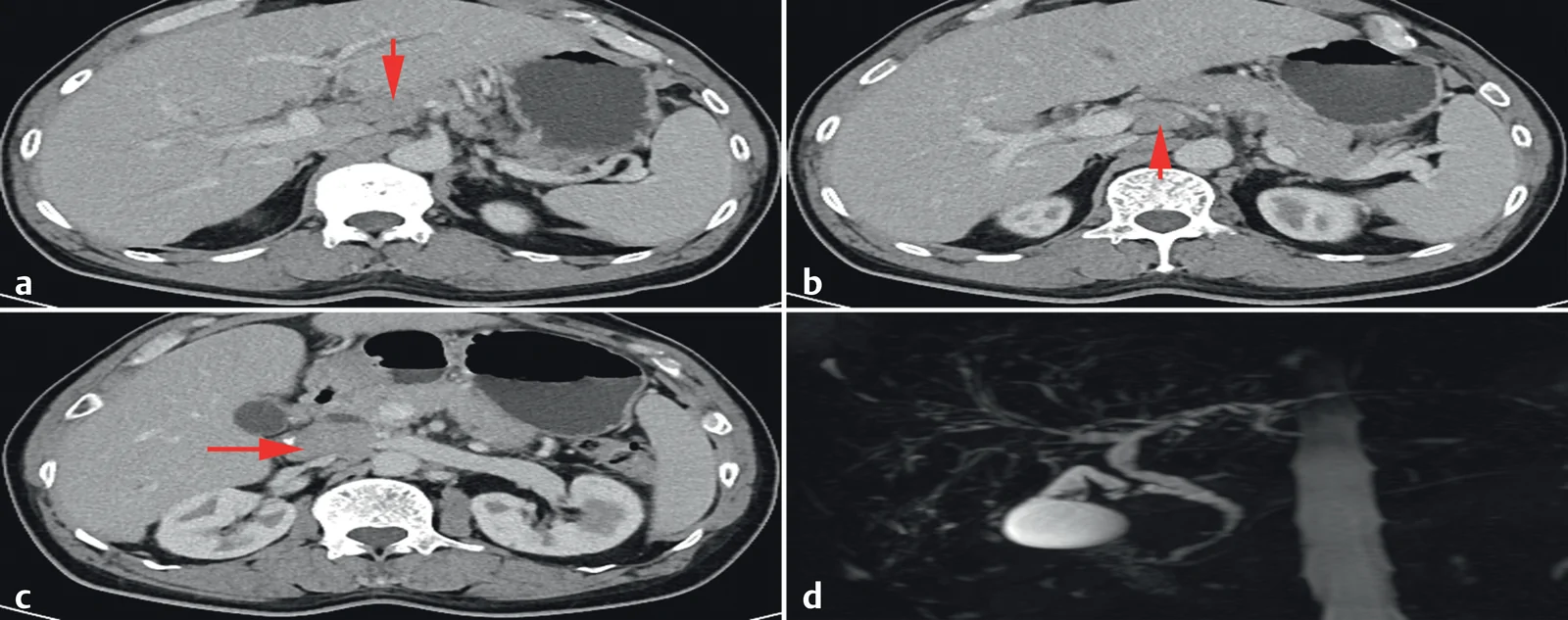
Abdominal contrast-enhanced CT and MRCP. ( **a** – **c** ) Contrast-enhanced CT images revealing multiple enlarged intra-abdominal lymph nodes. ( **d** ) MRCP demonstrating mild intrahepatic bile duct dilation. CT, computed tomography; MRCP, magnetic resonance cholangiopancreatography.

**Fig. 3:**
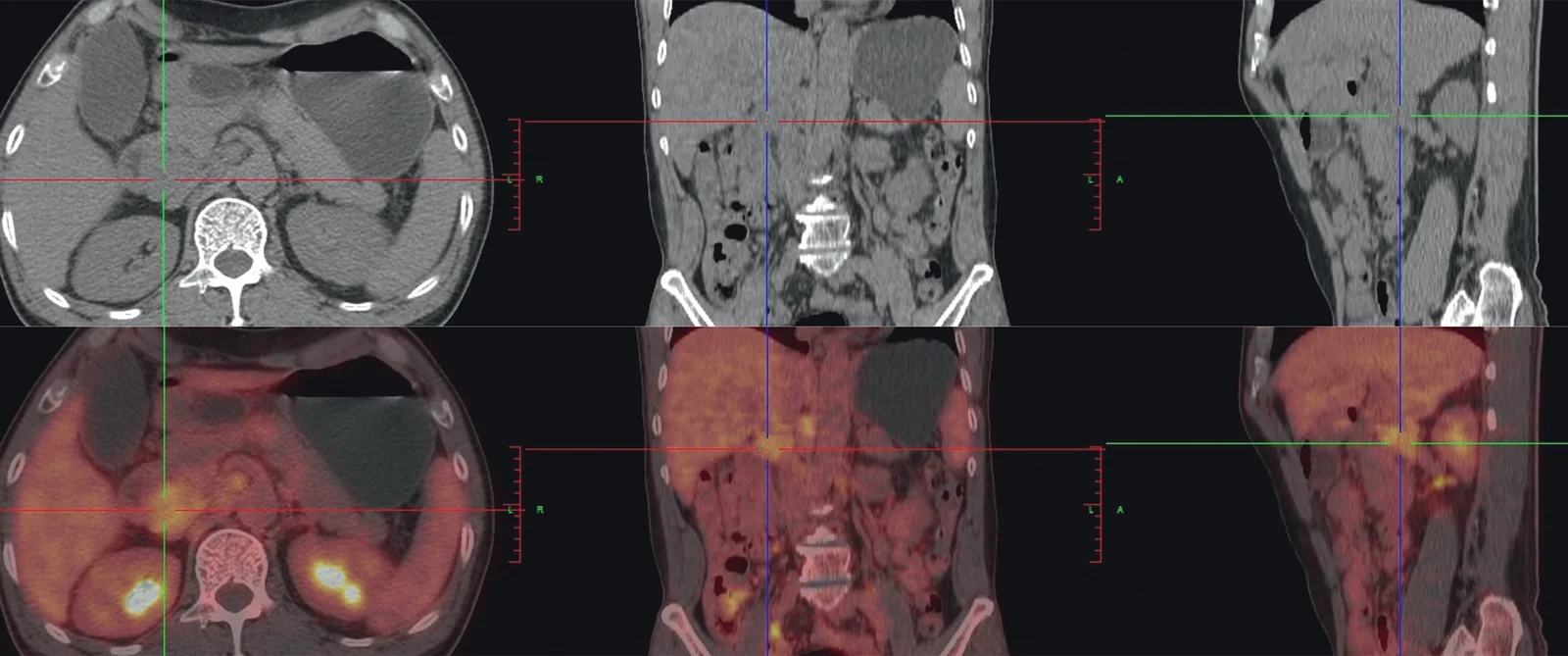
PET/CT. The PET/CT scan showing multiple enlarged lymph nodes with increased metabolic activity throughout the body ( SUV _max_ , approximately 6.08). The largest lesion is located posterior to the descending duodenum and measures approximately 17 mm in the short-axis diameter. PET/CT, Positron emission tomography/computed tomography; SUV _max_ , maximum standardized uptake value.

Endoscopic ultrasound (EUS) identified multiple enlarged intra-abdominal lymph nodes, with the largest (3.2×2.0 cm) located posterior to the descending duodenum, exhibiting a lobulated structure and abundant vascularity ( Fig. 4 ). Three passes of EUS-guided fine-needle biopsy were performed using a 22-gauge needle ( Video 1 ). Histopathological examination revealed lymphoid tissue with prominent eosinophilic infiltration (30/high-power field). Cytology was negative for malignancy, confirming the diagnosis of KD-associated abdominal lymphadenopathy ( Fig. 5 ).

**Fig. 4:**
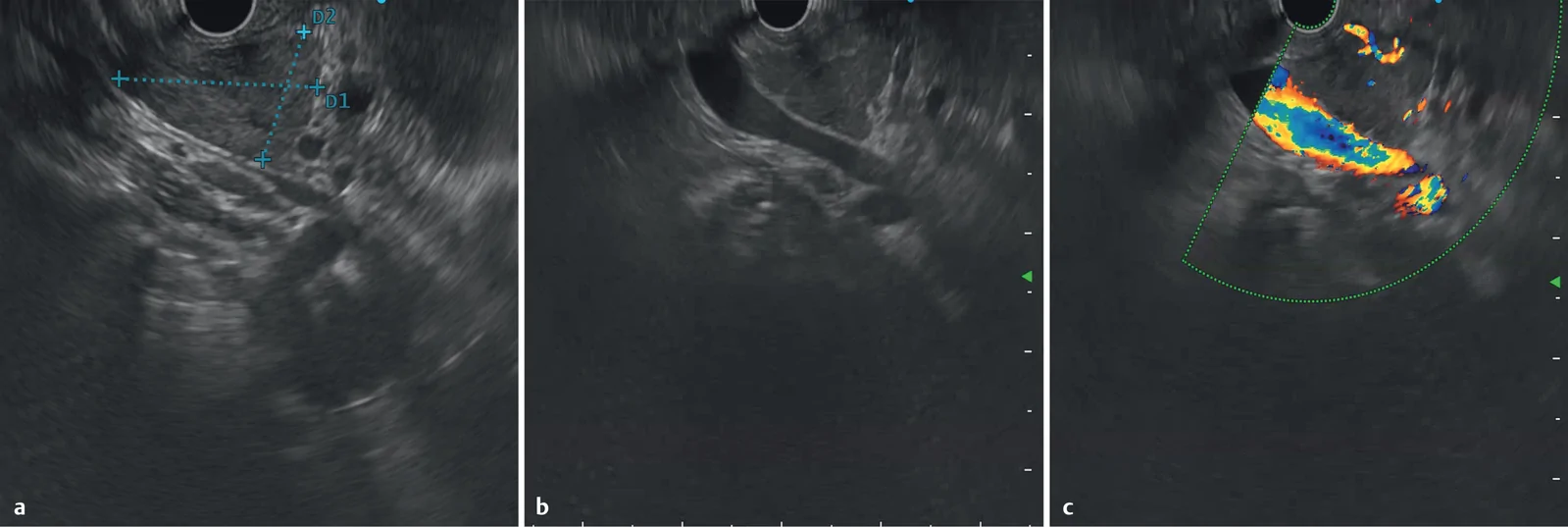
EUS assessment of enlarged intra-abdominal lymph nodes. ( **a** ) A lymph node located posterior to the descending duodenum, measuring approximately 3.2 × 2.0 cm. ( **b** ) Lobulated structure. ( **c** ) Abundant vascularity. EUS, endoscopic ultrasound.

**Video 1** EUS evaluation and EUS-FNB procedure targeting the enlarged lymph nodes posterior to the descending duodenum. EUS, endoscopic ultrasound; EUS-FNB, endoscopic ultrasound–guided fine-needle biopsy. URI:


**Fig. 5:**
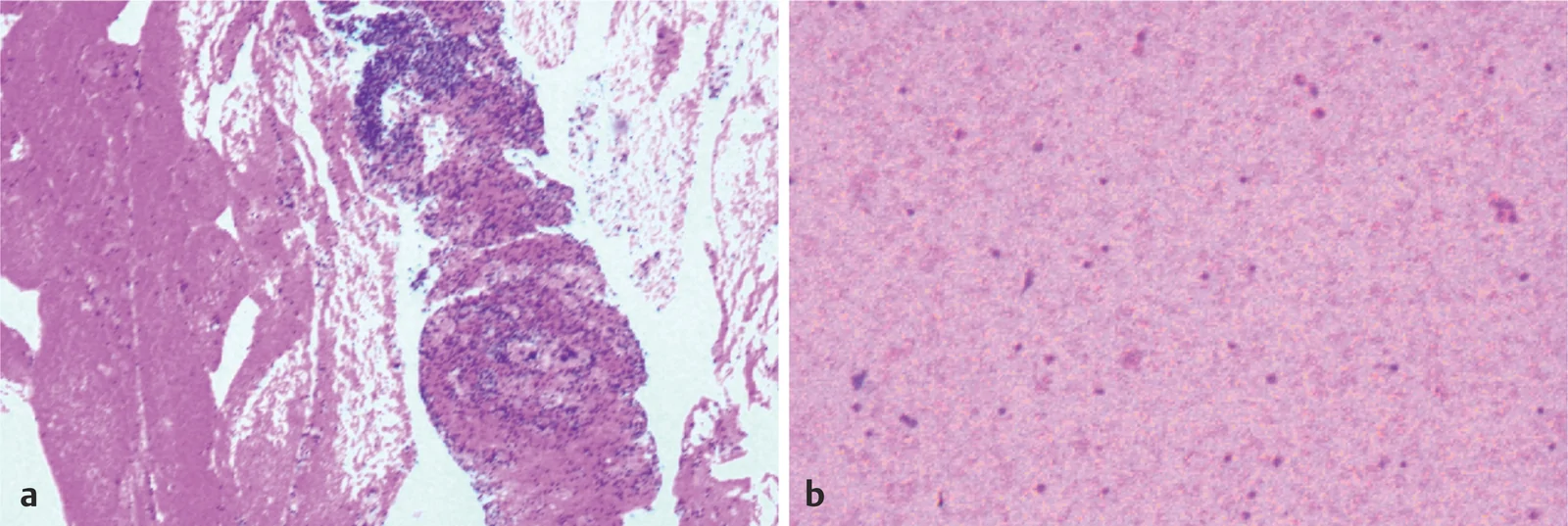
Pathological findings of EUS-FNB. ( **a** ) Histopathology showing lymphoid tissue with prominent eosinophilic infiltration (30 per high-power field). ( **b** ) Liquid-based cytology showing no malignant cells. EUS-FNB, endoscopic ultrasound–guided fine-needle biopsy.

To our knowledge, this is the first case report describing KD-associated abdominal lymphadenopathy evaluated by EUS and depicting its histopathological manifestations via EUS-guided biopsy. As KD is increasingly recognized as a systemic disease potentially associated with lymphoma, a long-term follow-up is essential. 4​ ​5​

Endoscopy_UCTN_Code_CCL_1AF_2AZ_3AD

## Data Availability

No new data were generated or analyzed for this article.

## Declaration of GenAI Use

The authors declare that no generative artificial intelligence tools were used in the preparation, writing, or editing of this manuscript.
